# Fair Healthcare Practices in Orthopedics Assessed with a New Framework

**DOI:** 10.3390/healthcare11202753

**Published:** 2023-10-17

**Authors:** Flaviu Moldovan, Liviu Moldovan

**Affiliations:** 1 Orthopedics—Traumatology Department, Faculty of Medicine, “George Emil Palade” University of Medicine, Pharmacy, Science, and Technology of Targu Mures, 540142 Targu Mures, Romania; 2 Faculty of Engineering and Information Technology, “George Emil Palade” University of Medicine, Pharmacy, Science, and Technology of Targu Mures, 540142 Targu Mures, Romania; liviu.moldovan@umfst.ro

**Keywords:** fair healthcare practice, sustainability, orthopedics, reference framework, healthcare facility, assessment

## Abstract

*Background and Objectives*: Healthcare systems are supported by the European ideology to develop their egalitarian concerns and to encourage the correct and fair behavior of medical staff. By integrating fair healthcare practices into sustainability, this requirement is addressed. In this research, our objective is to develop and validate, in the current activity of healthcare facilities, a new instrument for evaluating fair healthcare practices as a component of social responsibility integrated into sustainability. *Materials and Methods*: The research methods consist of deciding the domains of a new framework that integrates fair healthcare practices; the collection of the most recent fair healthcare practices reported by healthcare facilities around the world; elaboration of the contents and evaluation grids of the indicators; the integration of indicators related to fair healthcare practices in the matrix of the new framework for sustainable development; validation of the theoretical model at an orthopedic hospital. *Results*: The theoretical model of the new framework is composed of five domains: organizational management, provision of sustainable medical care services, economic, environmental, and social. The last domain is developed on the structure of the seven subdomains of the social responsibility standard ISO 26000. The seven indicators that describe fair healthcare practices are attitudes of the profession towards accreditation, effective intervention application, promoting a culture of patient safety, characteristics that affect the effectiveness of transfers, effective healthcare practices, feedback to medical staff, safety checklists. The new reference framework was implemented and validated in practice at an emergency hospital with an orthopedic profile. *Conclusions*: The practical implementation highlighted the usefulness of the new reference framework, its compatibility, and the possibility of integration with the reference frameworks for the evaluation of European hospitals, with the national legislation for the accreditation of hospitals and outpatient units, as well as with the ISO 9001 standard regarding the implementation of quality management systems. Its added value consists in promoting sustainable development by orienting staff, patients, and interested parties towards sustainability.

## 1. Introduction

In the last period, sustainability became a fundamental criterion in the evaluation of public health programs. Health is an investment that is itself sustaining and sustainable, and it is necessary to abandon conceptualizations of sustainability that focus on consumable medical interventions required to achieve health [1]. Exploring the specialized literature, we found that a series of studies present conceptual and practical developments regarding the integration of fair healthcare practices in sustainability.

Healthcare systems with egalitarian concerns are supported by the current European ideology of solidarity, human dignity, and equality. However, in many cases, these have proven to be unsustainable [2]. Equality and solidarity are closely related to the belief that all people have dignity. Hence, human dignity becomes the foundation of solidarity, equality, and, consequently, sustainable development. According to Ferdynus [3], in human dignity, there is a distinction between actual dignity, potential dignity, and existential dignity, and Ferdynus states that all human beings have existential dignity or potential dignity. The biomedical principle of justice, which was first formulated by Beauchamp and Childress, advocates that healthcare workers behave in a fair and just manner in relation to what they owe patients [4]. However, it is not detailed what obligation is imposed on healthcare workers from high-income countries to work in low- and middle-income countries in order to equitably distribute the availability of healthcare workers.

While economic processes solve the problem of scarcity through rationalization, which has the effect of increasing efficiency, ethics reflects the way of fair distribution of scarce goods. This necessarily includes the means of transparent and fair rationalization. Such a rationalization in a healthcare system cannot be achieved with non-medical criteria, such as the social function or the age of the patient, which are inadequate. Only medical criteria can bring sustainable solutions through quality-of-life measures that avoid extreme inequity [5]. Leung et al. [6] describe fair guidelines as a learning cycle in healthcare where evidence is rapidly generated and integrated into practice with digital support. They propose archiving data from different types of studies, such as clinical studies, meta-analyses, systematic reviews, etc. so that they can be quickly reproduced and to allow the continuous updating of medical evidence [7].

To practice “fairly and correctly”, a clinician must balance the needs of the few and the many: the individual patient in front of him and the other patients in the waiting room [8]. With this approach, medical staff must take into account the immediate clinical needs of current patients but also how their actions impact future patients. Through the existence of fair processes for the evaluation of medical technologies, relevant societal values can be captured that support the public reimbursement decisions for these technologies [9].

In practice, a limited number of validated instruments are accessible that favor the perception and measurement of aspects related to the sustainability of healthcare practices. Malone et al. [10] developed a clinical sustainability evaluation tool. It is designed to assess the factors that promote sustainable practices in clinical settings. Lennox et al. [11] show that healthcare sustainability does not have a unanimously accepted definition or a dedicated framework for its evaluation. His finding is based on the literature review in which the sustainability approach has highlighted 4 strategies, 16 models, 8 tools, and 32 reference frameworks.

Shigayeva and Coker [12] conclude that the conceptual proposals identified for the analysis of sustainability in healthcare systems do not have an explicit conceptualization of what a healthcare system is. Funders of public health programs are increasingly concerned about the sustainability of the changes they initiate. Although this requirement exists, Scheirer [13] shows that a widely used paradigm has not yet been developed through which research results can be accumulated in generalizable findings.

Starting from the previously presented gaps that were identified in the scientific literature, our study formulates the following research questions:

(RQ1): What are the fair healthcare practices reported in the scientific literature and validated in practice by representative medical institutions at the international level?

(RQ2): With the support of these practices, what indicators can be defined for the evaluation of fair healthcare practices?

(RQ3): How should we qualitatively and numerically define the indicators of a new reference framework so as to allow the evaluation and monitoring of progress in the implementation of good healthcare practices?

The main objective of this research is formulated on the basis of the research questions and consists of the development of a new, complex instrument for evaluating fair healthcare practices as a component of social responsibility, integrated into sustainability.

The secondary objective of the research is to provide assurance that this instrument can be integrated with accreditation legislation and other reference frameworks currently used in hospitals.

## 2. Materials and Methods

We used the following research methodology:

1. Defining the domains of the new sustainable development framework in which fair healthcare practices are integrated;
2. Studying scientific literature from relevant databases and extracting the most recent healthcare practices reported by healthcare facilities around the world;
3. Elaborating on the content and evaluation grids of the indicators that describe fair healthcare practices;
4. Integrating indicators related to fair healthcare practices in the matrix of the new framework for sustainable development;
5. Practically validating indicators related to fair healthcare practices at an orthopedic field hospital.

### The Reference Framework Areas

We started this research by establishing the domains of the new framework for sustainable development: Health–Sustainability (H-S). From the exploration of the scientific literature, we found that along with the 3 domains of sustainability: economic, environmental, social, to ensure the efficient functioning of the system, 2 additional domains were needed. The inclusion of processes for providing and ensuring the quality of medical services and management processes [14] ensures the commitment and involvement of the entire healthcare staff and the management of the organization [15]. This would facilitate good implementation and efficiency mainly due to the support from the top management and the staff of the organization.

**Figure 1:**
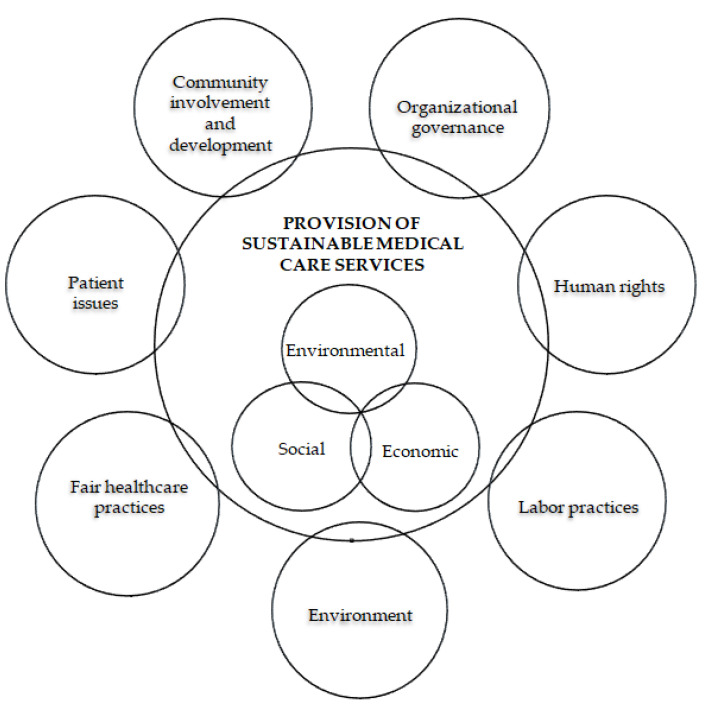
Health–Sustainability (H-S) reference framework conceptual model.

Therefore, the theoretical model of the H-S reference framework (Figure 1) consists of organizational management, provision of sustainable care services, economic, environmental, and social [16]. The last domain is treated in accordance with the 7 subdomains of the standard on social responsibility ISO 26000 [17]. These are adapted to medical specifics as follows: organizational_governance, human_rights, labor_practices, environment, fair healthcare practices, patient_issues, community_involvement, and development.

The conceptual model developed in this form is compatible with the reference frameworks for the evaluation of European hospitals DUQuE [18], the national legislation for the accreditation of hospitals [19] and ambulatory units [20], as well as with the ISO 9001 standard regarding the implementation of quality management systems [21].

**Figure 2:**
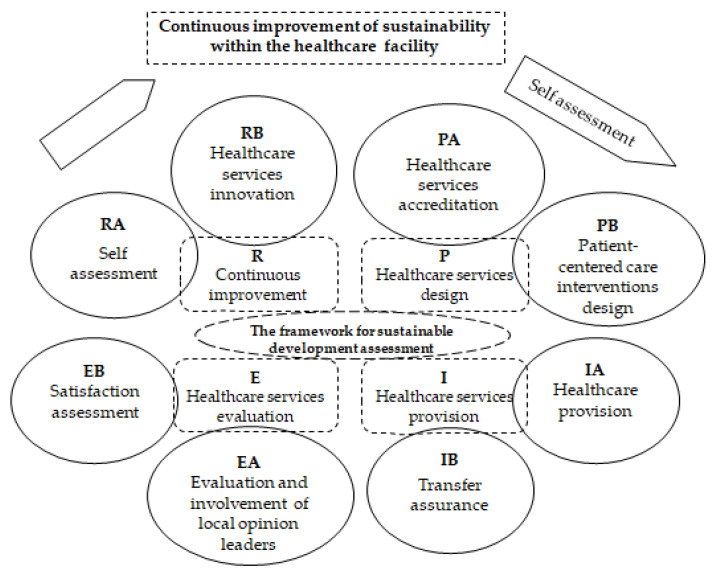
Succession and interconnection of basic medical activities in the quality cycle: P—plan, I—implement, E—evaluate, R—review.

In the next stage of this research, we designed the structure of medical care activities organized in the 4 stages of the quality cycle (Figure 2). In the P—plan (Healthcare service design) stage, we designed the activities (PA)—Healthcare services accreditation and (PB)—Patient-centered care interventions design. In stage I—implement (Healthcare service provision), the activities (IA)—Healthcare provision and (IB)—Transfer assurance were integrated. In the third stage E—evaluate (Healthcare service evaluation), we provided the activities (EA)—Evaluation and involvement of local_opinion leaders and (EB)—Satisfaction assessment. Respectively, in the last stage of R—review (Continuous improvement), the activities (RA)—Self-assessment and (RB)—Healthcare services innovation were carried out [16].

## 3. Results

### 3.1. Evidence of Fair Healthcare Practices in Healthcare Organizations

In the continuation of the research, we defined the content and evaluation grids of the indicators that make up fair healthcare practices.

After exploring relevant databases, such as Web of Science, PubMed, EMBASE (OVID), we extracted scientific papers that contain the most relevant concepts and descriptions for fair healthcare practices reported by hospitals around the world. Database exploration was performed using keywords like healthcare practices, fair, equity, justice, sustainability, and healthcare facilities. We preferred to select mainly recent articles published in the last 10 years. We included in this study only the articles that presented results confirmed by evidence, recent discoveries, clinical studies, or new knowledge. We compared the articles that described the same aspect and extracted the practices that were consistent with our study, the practices that allowed a greater degree of generality or a better traceability of the analyzed process.

At the end of this research stage, we obtained the most current fair healthcare practices, which were designed, tested, and implemented in the most representative hospitals in the world. The following sections present these contents used as input elements in the design of indicators that describe fair healthcare practices. We developed the corresponding indicators following the sequence of the quality cycle stages: P—plan (Healthcare_service design), I—implement (Healthcare_service provision), E—evaluate (Healthcare_service evaluation), respective R—review (Continuous improvement).

#### 3.1.1. Indicators for Healthcare Services Design

Oliveira et al. [22] analyzed managers’ and professionals’ perceptions of changes arising from accreditation in hospital management. They conclude that management changes in hospital organizations resulting from accreditation were broad, multiple, and consistent with improved service quality. The study by Ng et al. [23] shows that the gains of these programs may include increased staff commitment and communication, the formation of multidisciplinary teams, and positive changes in organizational culture. The metastudy conducted by Avia and Harivati [24] indicates that the benefits of accreditation have the effect of improving teamwork and productivity. Real quality improvements related to leadership, commitment, and support were also identified.

The study conducted by Andres et al. [25] concludes that the hospital accreditation process can contribute to changes in the staff’s perception of organizational culture, as there are different points of view on organizational culture between professional groups. The quality of care in the specialties of orthopedic traumatology [26] and ambulatory surgical care [27] in accredited hospitals is better than in non-accredited ones. This is mainly due to the improvement of the structure and organization of healthcare institutions.

Ehlers et al. [28] show that future attention should be paid to attitudes toward accreditation that are influenced by perceived difficulties in maintaining the system in relation to accreditation standards and data collection. Kakemam et al. [29] are of the opinion that quality improvement through hospital accreditation is a complex process with high demands on management and employees, which is influenced by the perceived level of bureaucracy, time consumption, and costs involved.

The previously presented medical practices are the input elements for defining the attitudes of the profession towards the accreditation indicator (Table A1 presents in detail the indicator PA5—Attitudes of the profession towards accreditation), which is part of the healthcare services accreditation basic medical activity.

Orthopedic trauma is an unforeseen event that often includes multiple fractures and amputations. This affects the patient’s way of life. In short-term recovery from trauma, patients can be helped by holistic approaches, pastoral care, coping skills, mindfulness, visiting colleagues, and educational resources [30]. Interventional strategies facilitate the reduction in negative psychological sequelae of major orthopedic trauma. These include longitudinal counseling, individual interventions, group interventions, and the possibility of early amputation [31].

In the case of people exposed to complex traumas, psychological interventions focused on trauma are effective for the management of comorbidities and mental health problems [32]. After treatment, evidence-based psychological interventions are effective in reducing symptoms of anxiety, depression, and post-traumatic stress disorder [33].

Wichman et al. [34] show that visits in which all health problems are analyzed are effective. For example, clinical examination of the hip requires a systematic approach to differentially diagnose hip problems with overlapping pain referred patterns. A comprehensive assessment, from deep to superficial, of the four main pain generators of the hip is required: the osteochondral, capsulolabral, musculotendinous, and neurovascular elements of the hip.

Providing disease-specific information improves patient perception. In this context, the insurance network, recommendations from doctors, the availability of programs, and the location of the practice are important for patients [35]. In their study, Wei and Chen [36] show that by making doctor–nurse–patient co-decisions, based on evidence, post-operative rehabilitation and restoration of ankle function are promoted in patients.

National and international societies of orthopedics and traumatology recommend geriatric cooperation models in traumatology [37]. These must be supported by the communication behavior of the medical personnel that is focused on the patient. The effects consist of the significant reduction in morbidity and mortality rates. Empathic skills of healthcare professionals are associated with better outcomes for patients [38].

The collection of patient-reported outcome measures is important to assess the safety and effectiveness of orthopedic treatments [39]. This requires healthcare professionals to use various data collection skills.

The previously presented medical practices are the input elements for defining the effective intervention application indicator (Table A3 presents in detail the indicator PB5—Effective intervention application), which is part of the patient-centered care intervention design basic medical activity.

#### 3.1.2. Indicators for Healthcare Services Provision

In ensuring basic safety, healthcare is a decade or more behind other high-risk industries [40]. A limited number of studies have evaluated the effectiveness of promoting organizational patient safety culture in improving healthcare performance. There is some evidence to suggest that organizational culture may be a relevant factor in nursing performance [41]. For example, educational sessions boost employee morale and can lead to improvements in work-related outcomes: attitude, job satisfaction, organizational commitment, and culture. In the study carried out by Rocha and Trevizan [42], it is shown that the healthcare service considers organizational culture a practical philosophy that must be implemented in the services under their responsibility and accepts the challenge of overcoming the barriers related to tradition, moving from discourse to practice.

Team training or tools that support team communication can lead to improved staff perceptions of safety culture, care processes, and better patient safety outcomes, such as decreased adverse events [43]. Managers’ visits to departments have the effect of improving staff perceptions and safety culture [43]. Most successful hospitals in changing institutional culture and reducing risk-standardized mortality revealed distinct patterns in conflict management capacity, authentic participation, and membership diversity [44].

Overall, all studies consider that there is little evidence to support the link between organizational culture and health performance [45]. Articulating the nature of this relationship is proving difficult [41]. Currently available evidence does not identify effective and generalizable strategies for changing organizational culture [46]. However, it is appreciated that investing in strategies that encourage high-performance organizational culture supports the efforts of hospitals to improve clinical results [47]. Such a result is indicated in the study carried out by Azar [48]. It shows that knee osteotomies performed in an outpatient setting were as safe as those performed in a hospital.

The previously presented medical practices are the input elements for defining the promoting a culture of patient safety indicator (Table A5 presents in detail the indicator IA5—Promoting a culture of patient safety), which is part of the healthcare provision basic medical activity.

Effective communication and planning are the most important factors in improving the transfer process and reducing adverse events [49]. Bracey et al. [50] showed that communication with a surgeon is the key factor for accurate assessment of the need for transfer to trauma centers.

The appropriateness of orthopedic transfers to a trauma center emergency department was studied by O’Connell et al. [51]. They found no relationship between transfer adequacy and insurance status or night/weekend transfers. Patients who have undergone transferred hip arthroplasties are at increased risk of readmission and medical complications within the first 90 days of care, which requires increased vigilance [52].

About half of the total annual economic burden for readmissions in the United States is medical and unrelated to the joint replacement procedure. Half of this is related to procedural complications [53]. These are affected by the multitasking of emergency department clinicians, the unpredictability of workload, difficulties in exchanging information between departments, the functional diversity of care teams.

Lack of critical care knowledge hinders effective communication. There is a tendency among community hospitals to inappropriately transfer uninsured patients with benign orthopedic injuries to high-level trauma centers. Better communication between hospitals and orthopedic surgeons can reduce inappropriate transfer of patients [54].

The transfer of orthopedic patients to tertiary care centers is determined by factors beyond the complexity of medical and surgical care [55]. Inpatient transfer and discharge interventions are believed to have measurable effects only in the long term, are effective only at higher intensities, and can only be quantified in certain subgroups of patients.

The previously presented medical practices are the input elements for defining the characteristics that affect the effectiveness of the transfers indicator (Table A7 presents in detail the indicator IB5—Characteristics that affect the effectiveness of transfers), which is part of transfer assurance basic medical activity.

#### 3.1.3. Indicators for Healthcare Services Evaluation

The demand for musculoskeletal care continues to grow rapidly, with more than 1.7 billion people globally suffering from musculoskeletal conditions [56]. Analysis of the results of the study carried out by Roy et al. [57] shows that “medical staff with professional skills” is the most important success factor in the quality management of hospital services. Changing the behavior of physicians and healthcare professionals requires more than authoritative teaching or traditional continuing medical education. Healthcare professionals can call upon opinion leaders to positively influence their colleagues and the clinical environment. However, it is advisable not to formalize their role in order not to dilute their professional influence [58].

The identification of opinion leaders based on personal characteristics and interpersonal networks is described by Holliday et al. [59]. There is some evidence that leaders’ time and work can influence the quality and safety of clinical outcomes, processes, and performance [60].

The metastudies carried out by Flodgren et al. [61,62] analyzed medical practices in 296 and 337 hospitals, respectively. They conclude that local opinion leaders, alone or in combination with other interventions, can successfully promote evidence-based medicine. The activity of local opinion leaders is more effective when combined with other complementary interventions, for example: reminders, audits and feedback, awareness visits, marketing strategies, local consensus processes, patient-mediated interventions.

The previously presented medical practices are the input elements for defining the effective healthcare practices indicator (Table A9 presents in detail the indicator EA5—Effective healthcare practices), which is part of the evaluation and involvement of local opinion leaders’ basic medical activity.

#### 3.1.4. Indicators for Continuous Improvement

The effectiveness of audits and feedback has been well-researched in studies based on experimental or quasi-experimental designs, which have demonstrated small and moderate but systematic effects on the effectiveness of professional improvements [63]. The process of giving and receiving feedback in orthopedic surgery training programs is unique compared to any other workplace [64]. When basic performance is low, feedback is more effective if the source is a colleague or a supervisor [65], and it is provided multiple times, both verbally and in written format [66]. It is necessary that it presents both explicit measures and an action plan [67].

A study by Brown et al. [68] regarding the theory of intervention on clinical performance feedback formulates three conclusions: (a) healthcare professionals and organizations have a finite capacity to interact with feedback; (b) their feedback interactions are influenced by their own beliefs about how patient care should be delivered; and (c) the most effective feedback is supported by clinical behaviors.

The previously presented medical practices are the input elements for defining the feedback to medical staff indicator (Table A11 presents in detail the indicator RA5—Feedback to medical staff), which is part of the self-assessment of basic medical activity.

Studies that have evaluated the effectiveness of patient safety checklists suggest some benefits in using them to improve protocol adherence and patient safety [69]. Studies have reported evaluations of checklists designed to improve surgical safety, medication prescribing, heart failure management, pain control, infection control precautions, and medical transfers. Studies have reported significant reductions in postoperative complications and medication problems and improved compliance with evidence-based medication prescribing, infection control precautions, and patient teaching procedures. In three studies, 30-day mortality was assessed and was significantly lower among patients assigned to the checklist-assisted group [70].

Implementation and application of checklists in prehospital emergency medicine have shown some benefits in improving guideline adherence and patient outcomes in airway management, patient records, identification and triage, and other prehospital interventions [71]. Surgical checklists have been associated with increased detection of potential safety risks, decreased surgical complications, and improved communication between operating personnel [72]. Surgical checklists, when implemented effectively, have the potential to be effective in reducing complication and mortality rates after surgery [73]. It is important that they are validated and refined before relying on them, as indicated in the study by Williams et al. [74] for supracondylar humerus fractures.

The previously presented medical practices are the input elements for defining the safety checklists indicator (Table A13 presents in detail the indicator RB5—Safety checklists), which is part of the healthcare services innovation basic medical activity.

### 3.2. Indicators Description and the Evaluation Model

Next, we designed the content of the indicators that make up the new reference framework. As we extracted the corresponding contents from the selected medical studies, we performed a detailed description of each indicator.

In order to facilitate the evaluation of the indicators, we have further elaborated a set of questions that cover their content. Through the answers received to these questions, the auditor evaluates the achievement degree of the indicator on a scale from 0 to 5: 0—not relevant, 1—low, 2—satisfactory, 3—good, 4—very good, 5—excellent.

Along with this, the auditor assesses the importance of each indicator for the healthcare facility, also on a scale from 0 to 5, as follows: 0—not relevant, 1—unimportant (subject of low importance for the organization), 2—reduced importance (the organization’s activity is compromised by non-compliance with this requirement), 3—important (the organization’s activity is affected by non-compliance with this requirement), 4—very important (healthcare coverage is jeopardized by non-compliance with this requirement), 5—high importance (the organization’s existence is compromised by non-compliance with this requirement) [16].

Considering the extensive content of the seven indicators that describe fair healthcare practices and the related evaluation grids, we presented them in Table A1, Table A2, Table A3, Table A4, Table A5, Table A6, Table A7, Table A8, Table A9, Table A10, Table A11, Table A12, Table A13 and Table A14 of Appendix A as follows: Table A1. The indicator PA5—Attitudes of the profession towards accreditation; Table A2. Scale for indicator PA5—Attitudes of the profession towards accreditation; Table A3. The indicator PB5—Effective intervention application; Table A4. Scale for indicator PB5—Effective intervention application; Table A5. The indicator IA5—Promoting a culture of patient safety; Table A6. Scale for indicator IA5—Promoting a culture of patient safety; Table A7. The indicator IB5—Characteristics that affect the effectiveness of transfers; Table A8. Scale for indicator IB5—Characteristics that affect the effectiveness of transfers; Table A9. The indicator EA5—Effective healthcare practices; Table A10. Scale for indicator EA5—Effective healthcare practices; Table A11. The indicator RA5—Feedback to medical staff; Table A12. Scale for indicator RA5—Feedback to medical staff; Table A13. The indicator RB5—Safety checklists; Table Scale for indicator A14. RB5—Safety checklists.

We exemplify the way in which the PA5—Attitudes of the profession towards accreditation indicator is defined in Table A1: The attitudes of the profession towards accreditation have an impact on its successful implementation. The attitudes of the profession towards accreditation are determined by beliefs regarding the positive impact of accreditation on quality, organizational performance, and collegial decision-making; perceived difficulties in maintaining the system in relation to accreditation standards and data collection; and the level of perception of bureaucracy, time consumption, and the costs involved. The questions formulated for its evaluation are as follows: Is a culture of quality created in the healthcare facility? Are staff consulted on the impact of accreditation on the quality of medical services provided? Is the impact of accreditation on the organization’s performance assessed? Are difficulties in data collection and system maintenance identified against accreditation standards? Are measures taken to reduce red tape, time consumption, and costs in accreditation activities? Are decisions made collegially?

The evaluation scale of the indicator PA5—Attitudes of the profession towards accreditation, presented in Table A2, consists of the scores: 1—Low: Staff consultations are periodically organized regarding the assessment of the impact of accreditation on the quality of the medical services offered; 2—Satisfactory: An organizational culture oriented towards quality is created in the healthcare facility. The values and mission of the healthcare facility are defined and, based on a plan, they are accepted, assumed, and promoted at the behavioral level by all members of the organization; 3—Good: Difficulties in data collection and system maintenance against accreditation standards are identified and corrective actions are formulated; 4—Very good: Measures are taken to reduce bureaucracy, time consumption, and costs in accreditation activities, and decisions are made collegially; 5—Excellent: The impact of accreditation on the organization’s performance is assessed, and an improvement plan is developed.

In continuation of the experimental research, we validated in practice the theoretical model elaborated by testing the new reference framework and the indicators that make it up at the Orthopedics Department of the Targu Mures County Emergency Clinical Hospital (CECHTM) [75].

**Figure 3:**
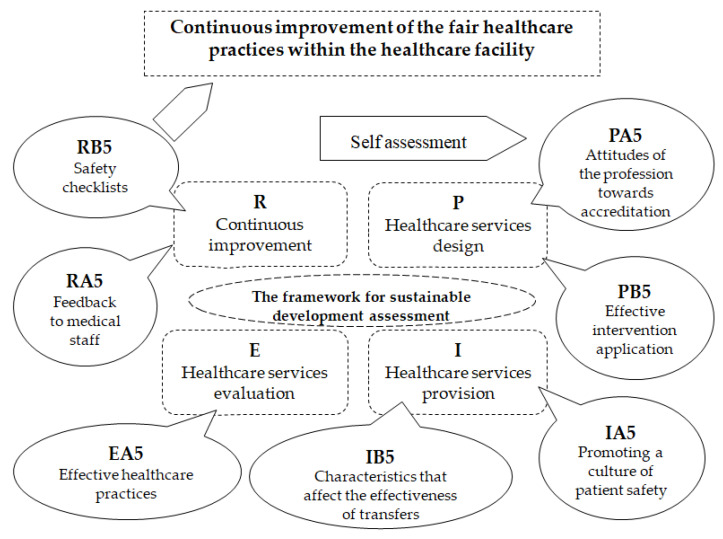
The fair healthcare practices continuous improvement cycle within the healthcare facility: P—plan, I—implement, E—evaluate, R—review.

Following the cycle of continuous improvement in Figure 3, which describes the sequence of indicators whose content and evaluation grids are presented in Table A1, Table A2, Table A3, Table A4, Table A5, Table A6, Table A7, Table A8, Table A9, Table A10, Table A11, Table A12, Table A13 and Table A14, we assessed the responsibility for fair healthcare practices. In the planning phase, the indicators PA5—Attitudes of the profession towards accreditation and PB5—Effective intervention application were employed. Then, we used the indicators IA5—Promoting a culture of patient safety and IB5—Characteristics that affect the effectiveness of transfers in the implementation phase. For the evaluation phase, the indicator EA5—Effective healthcare practices was employed. In the last phase, for review, the indicators RA5—Feedback to medical staff and RB5—Safety checklists were included.

### 3.3. Indicator Matrix

The matrix of indicators of the new H-S reference framework in Table 1 was developed based on the medical practices described in Section 3.1 for fair healthcare practices but also other previous research [16]. This connects the eight basic medical activities of the basic quality cycle (which are on the rows of the table) and the seven core subjects of social responsibility (which are on the columns of the table). The names of the projected indicators are suggestive so as to reflect this connection. Their content was developed following the identification in the scientific literature of a connection between a basic medical activity and a subject of social responsibility.

Where we have not discovered any successful activity in scientific literature, we have not designed indicators. This is the case of the link between the activity EB—Satisfaction assessment and core subject 5—Fair healthcare practices. The resulting H-S matrix has in its composition 57 indicators, of which 7 indicators describe the fair healthcare practices responsibility [76].

In the papers [16,76,77], the matrix of indicators is also presented, followed by the detailing of the indicators’ contents, the evaluation method, and the results regarding the practical implementation of the indicators that describe three core subjects of social responsibility: human rights, labor practices, and environment (Table 1, columns 2–4). In this research, we have studied the core subject of fair healthcare practices of social responsibility whose indicators are presented in column 5 of Table 1. The findings and results we obtained for the evaluation of the indicators that describe the fair healthcare practices of the emergency hospital are presented below.

PA5—Attitudes of the profession towards accreditation—CECHTM was placed in category IV, accredited with low confidence. Of the 9173 applicable indicators, 7997 indicators were compliant, and 1176 indicators were non-compliant, so a total percentage score of 85.59% was obtained, the percentage of indicators with a score (−10) being 23.37%. We identified difficulties in collecting data and keeping the system in line with accreditation standards. The hospital has a compliance plan for a period of more than 24 months, which demonstrates the high degree of compliance of the medical services provided with national accreditation standards and thereby the concern for patient safety.

Strengthening the culture of quality and safety in the hospital requires a clear understanding of how to monitor processes and outcomes and how to evaluate data and plan and implement continuous improvement by understanding and supporting the basic principles of quality management in healthcare services.

PB5—Effective intervention application—The psychology commission monitors the method of providing psychological assistance services to patients and hospital employees and takes measures to ensure the psychological support of patients in order to establish a psychodiagnosis, increase compliance with the medical act, and reduce anxiety regarding the medical act. During the year 2022, a number of 2396 patients were counseled. The patient’s psychological suffering is taken into account during medical interventions. Medical professionals empathize with patients, analyze all their health problems, and provide them with disease-specific information.

IA5—Promoting a culture of patient safety—Strengthening the hospital-level monitoring and evaluation capacity of healthcare-associated infections and antibiotic resistance is carried out by the Healthcare-Associated Infections Prevention Service. The annual plan for the prevention and control of this type of infection at the hospital level has been drawn up and distributed to departments and compartments in order to draw up their own plans for the prevention and control of nosocomial infections [78]. The microbial load was evaluated by collecting 7382 samples during bacteriological self-control, of which 87 samples exceeded the allowed limits.

Staff training measures have been ordered regarding the need to intensify current cleaning and disinfection by respecting the working concentration and contact time of disinfectant solutions. The persons identified with nasal portage were decolonized locally according to the antibiogram. For the inappropriate samples from the sterilettes, the instruments were properly processed, respecting the stages of disinfection, sterilization itself, handling, and keeping the sterilized material in appropriate conditions. In 2022, 728 cases of healthcare-associated infections were reported at the hospital level. For each of these, an epidemiological investigation was prepared, after which measures to prevent transmission were ordered.

In the present study, we identified the factors that contributed to the increase in the rate of confirmed healthcare-associated infections. We conducted interviews and held discussions with the specialized staff of CECHTM, from which we identified a wide variety of causes. First-order causes are, on the one hand, the people who participate in the medical act: patients, nurses, and doctors, and, on the other hand, the subsystems of the hospital: the information system, the clinical system, and the way in which the monitoring of hospital processes is ensured. Among the second-order causes, the elements that require the most attention and the highest priority approach are the awareness of missing information and the clinical surveillance procedure. We also identified an aspect related to the institutional culture, the lack of reporting incentives.

IB5—Characteristics that affect the effectiveness of transfers—The hospital ensures that there is communication between the staff performing the transfer, the staff from the shift changes, and the departments involved in the transfer of the patient by using the situation–history–assessment–recommendation technique. At the time of discharge, key care plan information is shared with the patient and the next healthcare provider. The development of communication skills is included in the continuous training plan for health personnel. Communication with the organizations that provide parallel medical assistance to the transferred patient is promoted.

EA5—Effective healthcare practices—During the visits carried out periodically through the departments, the local opinion leaders promote the use of good medical practice guidelines. When differing medical opinions arise, interventions are harmonized within comprehensive processes. Robotic technologies [79,80], computer technologies [81,82], and mechatronic rehabilitation systems [83] are used.

RA5—Feedback to medical staff—The evaluation of the individual professional performances of the medical staff is carried out on the basis of a procedure. This objectively assesses the professional performance of the contractual staff by directly comparing the degree and the way of fulfilling the requirements formulated by the job evaluation criteria. The evaluators are the heads of departments where the evaluated personnel work and the qualification awarded is approved by the hospital manager. The result of the evaluation is communicated individually, and the evaluated person signs the evaluation sheet.

RB5—Safety checklists—Surgical safety checklists are used in the format recommended by the World Health Organization (WHO) [84] and are adapted to local requirements and meet professional needs. They allow for ensuring the safety of patients but also the legal security of the medical staff. Their absence could generate liabilities for the hospital and the medical staff. A culture of clinical safety is created and reinforced among the medical staff based on the belief that safety lists are useful in increasing the quality of clinical services.

The values achieved for the indicators related to fair healthcare practices responsibility are registered in the self-assessment tool (Table 2).

**Figure 4:**
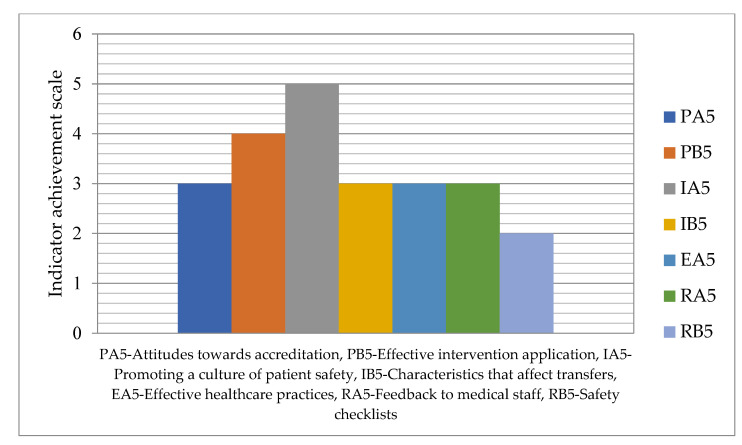
Achievement degree for the fair healthcare practices responsibility.

The degree of achievement of indicators related to fair healthcare practices is depicted in Figure 4 on a scale in the range of 1–5.

In this domain, the indicator RB5—Safety checklists has a minimum value of 2, while the highest value of 5 is recorded for the indicator IA5—Promoting a culture of patient safety.

**Figure 5:**
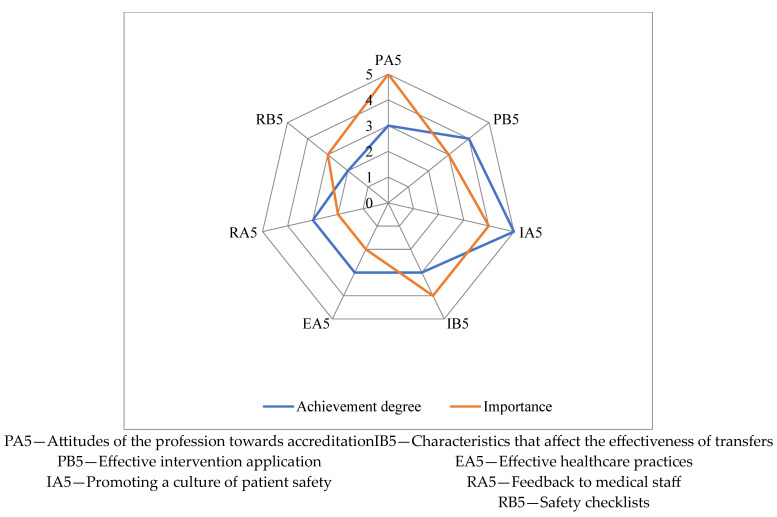
The fair healthcare practices evaluation graph.

The correlation between the importance and achievement degree of the indicators related to fair healthcare practices is depicted in the evaluation graph in Figure 5.

By adding the values of individual sustainability indicators from Table 2, the global sustainability indicator for fair healthcare practices (GS_FHP_) is obtained:

(1) $${GS}_{FHP}=\sum_{i=1}^{7}S_{i}=\sum_{i=1}^{7}I_{i}\cdot A_{i}=77$$

The greatest value for each indicator allows for computing the Global Sustainability for the fair healthcare practices at the maximum value (GSmax_FHP_):

(2) $${GSmax}_{FHP}=5\cdot \sum_{i=1}^{7}I_{i}=5\cdot 23=115$$

In this way, the overall fair healthcare practices sustainability level (LGS_FHP_) is computed as a percentage of the maximum value it can achieve:

(3) $${LGS}_{FHP}=\frac{{GS}_{FHP}}{{GSmax}_{FHP}}\cdot 100=\frac{77}{115}\cdot 100=66.95\%$$

The result obtained through this calculation indicates the extent to which the hospital fulfills the requirements related to fair healthcare practices.

**Figure 6:**
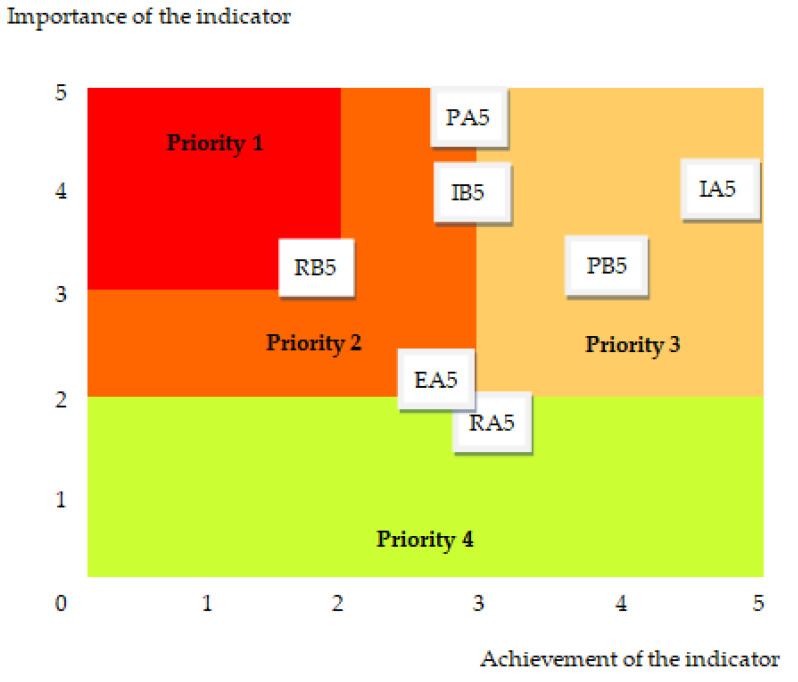
The assessment diagram for fair healthcare practices: PA5—Attitudes of the profession towards accreditation, PB5—Effective intervention application, IA5—Promoting a culture of patient safety, IB5—Characteristics that affect the effectiveness of transfers, EA5—Effective healthcare practices, RA5—Feedback to medical staff, RB5—Safety checklists.

In the continuation of the research, the results in an assessment diagram (Figure 6) were represented in order to use them in plans for improvement measures.

With the support of this graphic representation, depending on the quadrant in which the indicators are located, improvement measures can be planned with high priority (1) up to low priority (4).

In the current situation, in order to improve fair healthcare practices, the highest priority is provided to the indicator RB5—Safety checklists.

## 4. Discussion

Practice validation of the responsibility regarding fair healthcare practices, which make up the new H-S reference framework, was carried out at the CECHTM emergency hospital. The team of evaluators was composed of four auditors who had different responsibilities: head doctor of the orthopedic department, resident orthopedic doctor, chief assistant, and one responsible for quality assurance.

The unanimous opinion of the evaluators was that the indicators that describe fair healthcare practices have adequate content for the proposed purpose. They are in accordance with medical practices in international hospitals. There were situations in which some additions had to be made to the description of the indicators so that they adapt as best as possible to the particularities of the evaluated institution. From this perspective, interested users in applying this new evaluation system, at the beginning of the evaluation, should analyze the content of the indicators and then customize them to the concrete situation in which they are in. The development of a glossary with specific terminology would facilitate the mutual understanding of those involved in the evaluation.

In our study, we found that the content of the reference framework, through its indicators, is compatible and can be incorporated with the European framework DUQuE [18] but also with the national accreditation requirements [19,20]. In addition, the new framework has added value in directing the hospital towards sustainable development.

We have also found that planning the evaluation is very important in order to obtain the consensus of the evaluated persons regarding the inclusion in the planned time period. The chief auditor must be a person with authority and a good organizer who knows audit techniques. In general, the participation in this pilot program was appreciated as a success by all the members of the evaluation team, which gave satisfaction to the participants.

The pilot implementation of the new reference framework created the opportunity to increase responsibility regarding fair healthcare practices to promote responsible and sustainable behavior of healthcare personnel. The new reference framework allowed the analysis of medical practices from a complex approach, which favors the implementation of sustainable processes within the hospital.

The results of our study reveal that the RB5—Safety checklists indicator requires priority treatment. This means further testing and pilot validation of safety checklists to ensure that they contain all relevant elements and are consistently interpreted by users. There is a need for further training of medical staff on the correct use and compliance of the checklists. Over time, the effects of improving patient safety as a result of improving compliance in medical care processes will have to be highlighted. This can be evaluated by reducing the length of stay in intensive care or emergency rooms, reducing surgical complications, and improving the administration of antibiotics.

The results of this research highlight the superior quality of orthopedic trauma care at CECHTM, which is an accredited hospital, compared to other non-accredited hospitals in the Central Region of Romania. These findings are in agreement with the results of the study conducted by Greenfield and Braithwaite [26].

Similar to the findings of the study conducted by Kakemam et al. [29], we found that there is a high level of perception of bureaucracy, which is time-consuming. These aspects can be reduced by modernizing the IT system and simplifying some procedures.

Unlike the study by Wichman et al. [34] that supports the performance of consultations in which all health problems are analyzed, we found that in the click exam of the hip, a systematic approach is not carried out to diagnose all hip problems with overlapping pain referred patterns.

In our study, we found that there is a need for an improvement in the organizational culture of the medical assistance service, as also revealed by the study conducted by Rocha and Trevizan [42]. We could not highlight a direct link between the hospital’s organizational culture and the medical performance in orthopedics.

In agreement with the results reported by O’Connell et al. [51], we found a proper nature of interdepartmental and intrahospital transfers regardless of the period in which they were carried out, including at night or on weekends.

Opinion leaders are persons with professional authority, but contrary to the studies carried out by Flodgren et al. [61,62], we could not highlight the use of other complementary interventions with the exception of good medical practice guidelines.

In our study, we found that although the results of the evaluations performed are communicated to the staff of the orthopedics–traumatology department, they do not know the improvement measures that fall to them as a result of the evaluations performed. In contrast to the findings reported by Ivers et al. [63], this does not generate systematic effects regarding the effectiveness of professional improvements.

Although safety checklists are used for some medical procedures, they are not generalized (e.g., tibial plateau fractures) and are refined periodically, as indicated in the study by Williams et al. [74].

## 5. Limitations

This study has some limitations. Although the new reference framework created by the indicators that compose it provides an ensemble image of fair healthcare practices, they must be further specified according to organizational realities. From this perspective, not all the requirements of healthcare facilities are covered, and the reference framework can be expanded depending on the medical specialties of the hospital, the form of organization, and ownership. The validation of the reference framework was carried out at an emergency hospital with an orthopedic profile, which generates another limitation of the study and provides some implications for further research. The areas identified for further exploration include validating the indicators in other medical specialties. The indicators can be further expanded so that they still cover a large area in the medical field. This is where the future study directions come from, by diversifying the content of the indicators that should respond to the widest possible concerns in the medical field. Based on them, appropriate software tools can be developed to facilitate easy use of the system and the tracking of results and continuous improvement programs.

## 6. Conclusions

In this research, we presented the aspects of social responsibility related to fair healthcare practices. These are evaluated through seven indicators that are part of the new health–sustainability framework. A detailed description of the indicators and evaluation grids are provided. They have an innovative format that evaluates the couple importance-degree of achievement of the indicator. The indicators are designed by collecting medical practices from hospitals around the world, which are reported in medical studies. The evaluation methodology allows for establishing the performance levels of fair healthcare practices within the healthcare facility and directing the staff toward sustainable development.

The H-S reference framework is compatible and can be integrated with the requirements formulated by the national hospital accreditation legislation and with the European DUQuE hospital quality assessment framework. Practical validation of the H-S reference framework at an orthopedic hospital highlighted the adequacy of the proposed purpose and the fact that compared to the implemented referential, it promotes sustainable development.

## Figures and Tables

**Table 1:** Indicator matrix of the H-S framework.

	Social Responsibility	1—Organizational Governance	2—Human Rights	3—Labor Practices	4—Environment	5—Fair Healthcare Practices	6—Patient Issues	7—Community Involvement and Development
Quality Cycle								
(P) Healthcare services design	PA—Healthcare services accreditation	PA1—Decision structures and processes	PA21—Health care services accessibility PA22—Medical care services for disadvantaged groups	PA3—Promotion of change and professional development	PA4—Environ-mental impact plan	PA5—Attitudes of the profession towards accreditation	PA6—Performance information	PA7—Community involvement activities
	PB—Patient-centered care interventions design	PB1—Quality assurance processes design	PB2—Interventions with positive effects on patient satisfaction	PB3—Quality assurance of patient- centered medical interventions	PB4—Environ-mental criteria for selection of materials used in interventions	PB5—Effective interventions implementation	PB6—Patient self-care design and self-management	PB7—Content of the interventions adapted to the community
(I) Healthcare services provision	IA—Health care provision	IA1—Computerized support systems for clinical decisions	IA2—Specific medical approaches	IA31—Continuous healthcare education IA32—Practice guidelines employment and dissemination	IA41—Usage of recycled materials IA42—Waste recycling	IA5—Promotion of the patient safety culture	IA6—Critical features for improving the surveillance of patients with chronic conditions	IA71—Networking and partnership IA72—Involvement of volunteers and training networks
	IB—Transfer assurance	IB1—Transfer evaluation mechanisms	IB2—Fair transfer interventions	IB3—Interventions for transfers improvement	IB4—Environmen-tally friendly transfer interventions	IB5—Features that affect transfer effectiveness	IB6—Interventions to reduce problems in outpatients	IB7—Involvement and participation of professional associations
(E) Healthcare services evaluation	EA—Evaluation and involvement of local opinion leaders	EA1—Existence and recognition of local opinion leaders	EA2—Evaluation of current medical practices	EA3—Professional practices improvement	EA4—Improve-ment of environmental consumption	EA5—Effective work practices	EA6—Patient-specific issues management	EA7—Local opinion leaders involved in the community
	EB—Satisfaction assessment	EB1—Monitoring mechanisms assignment	EB2—Patient satisfaction degree	EB3—Medical staff satisfaction	Not relevant	Not relevant	EB6—Patient satisfaction degree regarding therapeutic benefits	EB7—Satisfaction regarding partnerships
(R) Continuous improvement	RA-Self assessment	RA1—Self-assessment tools	RA2—Freedom of expression assurance	RA3—Audit and feedback	RA4—Mechanisms for monitoring energy consumption and waste generation	RA5—Feedback to medical staff	RA6—Complaints management	RA7—Communitarian initiatives
	RB—Healthcare services innovation	RB1—Changes to healthcare services	Not relevant	RB3—Medical organization supported by Six sigma and Lean	RB4—Environmental measures	RB5—Safety checklists	RB6—Incident report	RB7—Educational visits

P—plan, I—implement, E—evaluate, R—review.

**Table 2:** Self-assessment tool for fair healthcare practices responsibility.

No.	Indicator Description	Importance (Ii)	Achievement (Ai)	Sustainability Indicator (Si = Ii·Ai)
1	PA5—Attitudes of the profession towards accreditation	5	3	15
2	PB5—Effective intervention application	3	4	12
3	IA5—Promoting a culture of patient safety	4	5	20
4	IB5—Characteristics that affect the effectiveness of transfers	4	3	12
5	EA5—Effective healthcare practices	2	3	6
6	RA5—Feedback to medical staff	2	3	6
7	RB5—Safety checklists	3	2	6

Ii—Importance, Ai—Achievement, Si—Sustainability Indicator.

## Data Availability

The data used in this study can be requested from the corresponding author.

## Appendix A

**Table A1:** The indicator PA5—Attitudes of the profession towards accreditation.

Indicator	PA5—Attitudes of the Profession towards Accreditation
Description	The attitudes of the profession towards accreditation have an impact on its successful implementation. The attitudes of the profession towards accreditation are determined by: - Beliefs regarding the positive impact of accreditation on quality, organizational performance, and collegial decision-making; - Perceived difficulties in maintaining the system in relation to accreditation standards and data collection; - The level of perception of bureaucracy, time consumption, and the costs involved.
Evaluation question	Is a culture of quality created in the healthcare facility? Are staff consulted on the impact of accreditation on the quality of medical services provided? Is the impact of accreditation on the organization’s performance assessed? Are difficulties in data collection and system maintenance identified against accreditation standards? Are measures taken to reduce red tape, time consumption, and costs in accreditation activities? Are decisions made collegially?

**Table A2:** Scale for indicator PA5—Attitudes of the profession towards accreditation.

Score [A]	Achievement	Content
0	Not relevant	—
1	Low	Staff consultations are periodically organized regarding the assessment of the impact of accreditation on the quality of the medical services offered.
2	Satisfactory	An organizational culture oriented towards quality is created in the healthcare facility. The values and mission of the healthcare facility are defined and based on a plan, they are accepted, assumed, and promoted at the behavioral level by all members of the organization.
3	Good	Difficulties in data collection and system maintenance against accreditation standards are identified and corrective actions are formulated.
4	Very good	Measures are taken to reduce bureaucracy, time consumption, and costs in accreditation activities, and decisions are made collegially.
5	Excellent	The impact of accreditation on the organization’s performance is assessed and an improvement plan is developed.

**Table A3:** The indicator PB5—Effective intervention application.

Indicator	PB5—Effective Intervention Application
Description	Applying consultation interventions that have clear positive effects on patients: - Interventions to detect psychological distress; - Visits where all health problems are analyzed; - Providing disease-specific information that improves patient perception. Application of consultation interventions that have mixed effects on patients: - Communication behavior (of medical personnel) focused on the patient; - Empathy skills of medical staff; - The use by medical personnel of various data collection skills; - Co-decision making (including patient involvement).
Evaluation questions	Do medical interventions consider psychological distress? Does the patient’s medical consultation look at all health problems? Is disease-specific information provided to the patient? Is the communication behavior of medical personnel focused on the patient? Do medical staff empathize with patients? Do the collected data involve the skills of healthcare professionals? Are medical decisions made with patient involvement?

**Table A4:** Scale for indicator PB5—Effective intervention application.

Score [A]	Achievement	Content
0	Not relevant	—
1	Low	At the patient’s medical consultation, the patient’s psychological suffering is also assessed, and this is taken into account during medical interventions.
2	Satisfactory	At the patient’s medical consultation, all his health problems are analyzed.
3	Good	After diagnosis, the patient is provided with information specific to the disease.
4	Very good	The communication behavior of medical personnel is focused on the patient, and they empathize with patients.
5	Excellent	Medical professionals use various data collection skills, and medical decisions are made through patient involvement.

**Table A5:** The indicator IA5—Promoting a culture of patient safety.

Indicator	IA5—Promoting a Culture of Patient Safety
Description	Promoting a positive patient safety culture in all departments of the healthcare facility, which ensures appropriate values, attitudes, and behaviors regarding patient safety, is an important strategy that supports the improvement of healthcare system performance. Different activities can be used, such as: - Educational sessions for the development of personal action plans regarding employee behavior at work, employee well-being, job satisfaction, organizational commitment; - Introducing actions (for example: administrative) to change the organizational culture regarding the specific safety behavior (for example: the frequency of hand washing and the rate of nosocomial infections); - Team training: a package of structured methods for optimizing teamwork processes (for example: communication, cooperation, collaboration, and leadership); - Representative function support visits: Managers or senior leaders visit frontline patient care areas to observe and discuss current or potential threats to patient safety, as well as to support frontline staff in addressing these threats. It is assumed that healthcare facilities have their own cultures that are related to performance, and the interventions carried out provide a useful return on investment.
Evaluation questions	Are there cultivated values, attitudes, and behaviors regarding patient safety? Are educational sessions organized for the development of personal action plans regarding employee behavior at work, employee well-being, job satisfaction, organizational commitment? Are organizational culture change actions (e.g., administrative activities) planned regarding specific safety behavior (e.g., hand washing frequency and nosocomial infection rate)? Is team training provided that contains packages of structured methods for optimizing teamwork processes? For example: Communication, cooperation, collaboration, and leadership are assessed. Are representative function support visits conducted where managers or senior leaders visit frontline patient care areas to observe and discuss current or potential patient safety threats? During the visits, are frontline staff supported in addressing these threats? Is patient safety culture a component of organizational culture?

**Table A6:** Scale for indicator IA5—Promoting a culture of patient safety.

Score [A]	Achievement	Content
0	Not relevant	—
1	Low	A positive culture that ensures appropriate patient safety values, attitudes, and behaviors is promoted in all departments of the healthcare facility.
2	Satisfactory	Educational sessions are organized for the development of personal action plans regarding employee behavior at work, employee well-being, job satisfaction, and organizational commitment.
3	Good	To promote a culture of patient safety, actions aimed at changing the organizational culture regarding specific safety behavior are provided. Such actions can be administrative in nature, such as the frequency of hand washing, the rate of nosocomial infections, etc.
4	Very good	In order to promote a culture of patient safety, team trainings containing packages of structured methods for optimizing teamwork processes are provided. Patient safety culture is a component of organizational culture.
5	Excellent	To promote a culture of patient safety, representative function support visits are conducted where managers or senior leaders visit frontline patient care areas to observe and discuss current or potential patient safety threats. The patient safety culture is part of the healthcare facility’s strategy that supports improving the performance of the healthcare system.

**Table A7:** The indicator IB5—Characteristics that affect the effectiveness of transfers.

Indicator	IA5—Characteristics that Affect the Effectiveness of Transfers
Description	Characteristics that may hinder or complicate the effectiveness of transfer interventions and need to be assessed are: - Multitasking of emergency department clinicians; - The unpredictability of the workload (for example, in a recovery room), which makes it difficult to plan the availability of staff; - Difficulties in exchanging information between departments; - Lack of critical care knowledge prevents effective communication; - Functional diversity of care teams. - Intrahospital transfer and discharge interventions are possible: - Have measurable effects only in the long term (for example after 3 months); - Can be quantified only in certain subgroups of patients; - To be effective only at higher intensities.
Evaluation questions	Is multitasking of emergency department clinicians evaluated in the effectiveness of handover interventions? Is the influence of workload unpredictability on staff availability planning assessed? For example, recovery rooms are evaluated. Are the difficulties that arise in the exchange of information between departments assessed? Is the extent to which effective communication is hindered by lack of critical care knowledge assessed? Is the functional diversity of care teams ensured? Are longer-term in-hospital and outpatient transfer interventions evaluated to have measurable effects? For example, the effects are evaluated after 3 months. Are the effects of transfers quantified in specific patient subgroups in which data can be obtained? Are the effects of transfers evaluated for higher intensities that allow relevant data to be collected?

**Table A8:** Scale for indicator IA5—Characteristics that affect the effectiveness of transfers.

Score [A]	Achievement	Content
0	Not relevant	—
1	Low	Critical care knowledge gaps that prevent effective communication, as well as difficulties in sharing information between departments, are assessed, and steps are taken to ensure that these do not impede or complicate the effectiveness of handover interventions.
2	Satisfactory	Unpredictability of workload is assessed, and functional diversity of care teams is ensured through appropriate planning of staff availability.
3	Good	Multitasking of emergency department clinicians is assessed, and steps are taken to ensure that it does not impede or complicate the effectiveness of handover interventions.
4	Very good	The time period after which the effects of intrahospital transfer and discharge interventions can be measured is determined.
5	Excellent	Patient subgroups for which intrahospital transfer interventions can be quantified are determined. The effectiveness of in-hospital transfer and discharge interventions is evaluated and the intensity for which they are effective is determined.

**Table A9:** The indicator EA5—Effective healthcare practices.

Indicator	EA5—Effective Healthcare Practices
Description	The work of local opinion leaders is more effective when combined with other complementary interventions, for example: - Reminders; - Audits and feedback; - Awareness visits; - Marketing strategies; - Local consensus processes; - Interventions mediated by patients.
Evaluation questions	Is the activity of local opinion leaders combined with other complementary interventions? Are the following evaluated, for example: reminders; audits and feedback; awareness visits; marketing strategies; local consensus processes; patient-mediated interventions.

**Table A10:** Scale for indicator EA5—Effective healthcare practices.

Score [A]	Achievement	Content
0	Not relevant	—
1	Low	The activity of local opinion leaders is combined with reminders.
2	Satisfactory	The activity of local opinion leaders is combined with outreach visits.
3	Good	The activity of local opinion leaders is combined with local consensus processes.
4	Very good	The activity of local opinion leaders is combined with audit and feedback activities that allow for the formulation of corrective actions.
5	Excellent	The activity of local opinion leaders is included in marketing strategies and has the effect of improving healthcare services.

**Table A11:** The indicator RA5—Feedback to medical staff.

Indicator	RA5—Feedback to Medical Staff
Description	Communicating the results of the evaluation to all employees guarantees responsibility, transparency, and honesty in the organization, which is a key factor for achieving correct institutional practices.
Evaluation questions	How are the results of self-assessments communicated to all staff?

**Table A12:** Scale for indicator RA5—Feedback to medical staff.

Score [A]	Achievement	Content
0	Not relevant	—
1	Low	The results of the evaluations are communicated verbally to the heads of departments.
2	Satisfactory	The results of the evaluations are communicated in writing to the heads of departments.
3	Good	The results of the evaluations are communicated by the heads of departments to the subordinate staff.
4	Very good	All staff are informed of the evaluations results and are aware of the improvement tasks that fall upon them as a result of the evaluations carried out.
5	Excellent	Communicating assessment results to all employees ensures accountability, transparency, and honesty in the healthcare facility and ensures fair institutional practices.

**Table A13:** The indicator RB5—Safety checklists.

Indicator	RB5—Safety Checklists
Description	Safety checklists, also known as medical checklists, are a tool for improving care processes and patient safety outcomes. Safety checklists can vary in structure, content, and method of implementation. The safety checklists suggest some improvements in patient safety resulting from their use by healthcare teams, particularly in terms of: - Compliance improvements in some healthcare processes; - Reduction in intensive care or emergency room stays; - Reduction in surgical complications; - Improving the administration of antibiotics. The effectiveness of checklists increases if: - The design and implementation method is based on an evidence-based approach; - The checklist is pilot tested and validated (to ensure that the checklist contains all relevant elements and is interpreted consistently by users); - Staff members are trained on the correct use and compliance of checklists.
Evaluation questions	Are safety checklists used in medical practice? Is the design and implementation of safety checklists based on an evidence-based approach? Are checklists pilot tested and validated prior to actual use to ensure that the checklists contain all relevant elements and are consistently interpreted by users? Is medical staff trained in the correct use and compliance of checklists? What is the frequency of training?

**Table A14:** Scale for indicator RB5—Safety checklists.

Score [A]	Achievement	Content
0	Not relevant	—
1	Low	Safety checklists are used in medical practice.
2	Satisfactory	An evidence-based approach is applied to the design and implementation of safety checklists.
3	Good	Before actual use, checklists are pilot tested and validated to ensure they contain all relevant elements and are consistently interpreted by users.
4	Very good	Medical staff members are frequently trained in the proper use and compliance of checklists.
5	Excellent	The use of safety checklists results in improvements in patient safety as a result of improved compliance in healthcare processes, reduced lengths of stay in intensive care or emergency rooms, reduced surgical complications, improved antibiotic administration.
